# Comparison of Self-Tracking Health Practices, eHealth Literacy, and Subjective Well-Being Between College Students With and Without Disabilities: Cross-Sectional Survey

**DOI:** 10.2196/48783

**Published:** 2024-04-10

**Authors:** Soyoung Choi

**Affiliations:** 1 Department of Kinesiology and Community Health University of Illinois Urbana-Champaign Urbana, IL United States

**Keywords:** college students, personal health data, self-tracking, eHealth literacy, well-being, tracking, students, disability, cross-sectional survey, pediatric care, adult care, smartphone health app, application, literacy

## Abstract

**Background:**

College students with disabilities need to transition from pediatric-centered care to adult care. However, they may become overwhelmed by multiple responsibilities, such as academic activities, peer relationships, career preparation, job seeking, independent living, as well as managing their health and promoting healthy behaviors.

**Objective:**

As the use of smartphones and wearable devices for collecting personal health data becomes popular, this study aimed to compare the characteristics of self-tracking health practices between college students with disabilities and their counterparts. In addition, this study examined the relationships between disability status, self-tracking health practices, eHealth literacy, and subjective well-being among college students.

**Methods:**

The web-based questionnaire was designed using Qualtrics for the cross-sectional online survey. The survey data were collected from February 2023 to April 2023 and included responses from 702 participants.

**Results:**

More than 80% (563/702, 80.2%) of the respondents participated voluntarily in self-tracking health practices. College students with disabilities (n=83) showed significantly lower levels of eHealth literacy and subjective well-being compared with college students without disabilities (n=619). The group with disabilities reported significantly lower satisfaction (*t*_411_=–5.97, *P*<.001) and perceived efficacy (*t*_411_=–4.85, *P*<.001) when using smartphone health apps and wearable devices. Finally, the study identified a significant correlation between subjective well-being in college students and disability status (*β*=3.81, *P*<.001), self-tracking health practices (*β*=2.22, *P*=.03), and eHealth literacy (*β*=24.29, *P*<.001).

**Conclusions:**

Given the significant relationships among disability status, self-tracking health practices, eHealth literacy, and subjective well-being in college students, it is recommended to examine their ability to leverage digital technology for self-care. Offering learning opportunities to enhance eHealth literacy and self-tracking health strategies within campus environments could be a strategic approach to improve the quality of life and well-being of college students.

## Introduction

Youths or young adults with disabilities related to vision, hearing, mobility, and cognitive ability often experience functional limitations when performing self-care and daily activities [1]. Moreover, in addition to physical or sensory challenges, they often encounter limited job opportunities and employment prospects in the early stage of their career, in comparison with their peers without disabilities [2]. Recent rehabilitation interventions and programs have focused on optimizing their perceived quality of life including a sense of personal control over an individual’s life course and satisfaction with personal development [3]. To establish basic self-care skills, diverse health education programs are offered in community and education settings [4]. According to Orem, self-care refers to the practice of activities that individuals initiate and perform on their own behalf in maintaining life, health, and well-being [5]. To ensure a successful transition from pediatric-centered care to adult care, it is necessary to develop and establish optimized self-care strategies during the initial phase of young adulthood [6].

With the advancement of health information and digital health technology, an increasing number of laypeople engage in self-tracking health and share their personal health data with family members, friends, and health care providers using mobile, Internet-connected devices such as smartphone apps and wearable sensors [7]. Self-tracking health represents the repeated measurement and recording of health-related information about oneself for self-knowledge of health status, health behavior change, and health monitoring [8]. Recent digital health technology allows individuals to gather personal health information in real-life situations and engage in remote health interventions by sharing health data with health care providers [9]. A previous study revealed that young adults (N=16) aged 18 years and older showed a preference for mobile health app features that include setting and tracking health behavior goals, receiving feedback and advice on health behavior change, alerts and reminders, and adequate privacy settings [2]. Virella Pérez et al [10] mentioned that the use of digital health technology has the potential to assist young adults in self-care during the transition to adult care; however, the available evidence on its usability, feasibility, generalizability, and effectiveness is still lacking.

Despite the low cost, convenience, and automated self-tracking health features offered by digital health or mobile technology, we still have an insufficient understanding of its impact on health behavior changes in young adults with disabilities. Since users’ technology adoption and continued use are influenced by their distinct needs, goals, expectations, usefulness, efficacy, perceived ease of use, satisfaction with features, and context [11], it is possible that consumer technology released in the market may not adequately address the functional limitations of this particular group. For instance, the visually oriented designs of smartphone health apps may not provide full accessibility for users with low vision or blindness, and the limited keypad space on the screens of smartphones can be inconvenient for individuals with dexterity-related challenges. The hypothesis of this study posited that the experiences with self-tracking health among young adults with disabilities are different from those without disabilities.

The World Health Organization (WHO) [12] highlighted that health literacy is an important factor for preventing chronic illnesses because it significantly affects one’s capability against chronic illnesses, including addressing risk factors [13]. Health literacy refers to the degree to which individuals can find, understand, and use information and services to inform health-related decisions and actions for themselves and others [14]. Poor health literacy affects negative health outcomes as well as reduced satisfaction with health care services [15]. Recently, in the digital era, the concept of eHealth literacy, also known as digital health literacy, has emerged to assess individuals’ competence in using the Internet to navigate health care resources and access health information [16]. Based on a systematic review conducted on eHealth literacy among college students, a considerable number of adults were extensively engaged with the Internet and expressed significant confidence in their ability to search for electronic health information [17]. Nevertheless, the review also discovered conflicting outcomes, exposing cases of unsophisticated health information-seeking and a lack of proficient critical appraisals while evaluating the searched health information. Recognizing the discordance between perceived and evaluated eHealth literacy among college students, this study examined and compared the eHealth literacy levels of college students, both with and without disabilities.

During the global COVID-19 outbreak, there was a significant increase in the prevalence of mental health issues among college students [18]. Consequently, counseling centers on campuses took proactive measures to provide mental health care services, with the goal of improving the well-being and overall quality of life of college students [19]. Lattie et al [20] conducted a systematic review to investigate the effectiveness of digital mental health interventions for college students experiencing low levels of psychological well-being. The review not only examined the impact of digital health interventions on psychological outcome variables but also discussed the potential advantages of enhancing health care accessibility and cost-effectiveness [20]. Although some scholars have highlighted the negative impact of mobile phone addiction on this younger generation [21,22], strategically planned use of mobile health apps and wearable sensors could enhance the subjective well-being of college students [23].

Given the rise of innovative digital health technology and the increasing interest in personal health data, it is still unclear whether college students with disabilities experience disparities in digital health care and utilization, particularly in the context of mobile health technology. To fill this gap in prior research, the findings of this study were expected to contribute to the current understanding of self-tracking health practices among college students with disabilities. The conclusion of this study involves a discussion on future directions with a focus on leveraging mobile health technology and interventions to enhance subjective well-being and overall health among college students with disabilities. The potential benefits and implications of utilizing such technologies are narrated, emphasizing the promising role they can play in managing chronic conditions and preventing secondary health problems in younger populations with disabilities.

## Methods

### Survey and Participants

A cross-sectional online survey was conducted to examine the self-tracking health practices of college students, both with and without disabilities, using the Checklist for Reporting Results of Internet E-Surveys (CHERRIES) [24]. The web-based questionnaire was designed by the author using Qualtrics [25] and was reviewed for readability and clarity by 5 college students—2 college students without disabilities and 3 college students with disabilities—before being revised. The average time to complete the pilot survey was 15.4 minutes. Prospective participants representing college students with and without disabilities were invited to take part via email and social media platforms, including Twitter and Facebook. Upon clicking the URL in the email or social media post, participants were screened for eligibility and asked to provide informed consent. To be eligible for the survey, participants had to (1) be a US resident, (2) be aged between 18 years and 35 years, (3) be currently enrolled in college or university, (5) not have a cognitive impairment or psychiatric disorder, and (6) speak and write English. To assess disability status, the following additional screening question was asked: “Do you have a disability? If yes, please specify the type of disability you have (eg, visual impairment, hearing loss, mobility, learning).” No identifiable data, except for the email addresses of the participants for the compensation procedure, were collected. The online survey was open to respondents from February 20, 2023, to April 15, 2023. Participants who completed the survey were entered into a raffle for compensation in the form of Amazon eCodes. In 2 groups, 30 people each received US $20 worth of Amazon eCodes.

### Web-Based Data Collection

In this study, the self-tracking health practices refer to utilizing digital health technologies such as smartphone health apps, wearable sensors (eg, fitness trackers, smartwatches), and smart medical devices to regularly check one or more health-related data point as well as recording health data with manual tools (eg, pencil and notebook). The following questions were included in the survey to understand the self-tracking health practices and preferences of college students with and without disabilities: (1) “Do you currently track your health (eg, monitoring body weight, calculating calories intake, counting steps)?” (2) “What aspects of your health do you routinely track?” (3) “What types of tracking tools do you use to record your health data?” (4) “How frequently do you check your health data?” (5) “What are the reasons that you do not track your health?” (6) “What kinds of health data do you want to track in the future?” (7) “What smartphone apps have you used to manage your health?” (8) “What features do you prefer the health-related smartphone apps to contain?” (9) “What kinds of wearable devices have you used to manage your health?” (10) “Please describe any barriers or challenges you faced when tracking your personal health data.”

In this study, 2 self-reported questionnaires were used. First, the level of eHealth literacy was measured using the eHealth Literacy Scale (eHEALS) [26]. The eHEALS was developed to assess eHealth literacy for a wide range of populations [26]. It has 6 core skills such as traditional literacy, health literacy, information literacy, scientific literacy, media literacy, and computer literacy [27]. This self-report instrument includes 8 items and uses a 5-point Likert scale ranging from “Strongly disagree” to “Strongly agree.” Higher scores indicate higher levels of eHealth literacy. In the original study, which targeted a youth population aged between 13 years and 21 years, the internal reliability (Cronbach alpha) was .88, and the test-retest reliability (*r*) ranged from 0.40 to 0.68 [26]. In this study, the reliability (Cronbach alpha) was .88. Second, to quantitatively assess the perceived well-being of college students, the Flourishing Scale (FS) [28] was used. The FS is an 8-item, self-report scale and measures respondents’ perceived success in relationships, self-esteem, purpose, and optimism. It uses a 7-point Likert scale ranging from “Strongly disagree” to “Strongly agree.” Higher scores indicate higher functioning in social-psychological prosperity. The reported Cronbach alpha is .87, and the test-retest reliability is (*r*) 0.71. The reliability (Cronbach alpha) of this study was .91.

### Statistical Analysis

The minimum sample size of this study was 92 based on the number of predictors (n=5), targeted adjusted *R*^2^ of 0.30 with type 1 error of <.05, medium effect size of 0.3, and power of 0.8. Descriptive statistics and multiple regression analysis were used to investigate the characteristics and relationships between variables using R software (RStudio). To identify any detectable differences between continuous variables, independent *t* tests, ANOVAs, and Wilcoxon-Mann-Whitney tests were conducted. In the cases of counts or frequencies of categorical variables, chi-square tests were performed. Multiple linear regression analysis was conducted to examine the relationships between the main variables (ie, disability status, self-tracking health practice, eHEALS, and FS). For this regression model, the categorical variables (ie, doing self-tracking health, living with disability) were coded into 2 categories (ie, 0=No, 1=Yes).

### Ethical Considerations

Ethical approval for the survey was obtained from the University of Illinois Urbana-Champaign (Protocol ID: 23316), and the study was monitored by the Institutional Review Board of the University of Illinois Urbana-Champaign. Participants gave their informed consent prior to participating in the survey. The study data were anonymized and stored securely at the university. Through a raffle, 30 participants were randomly selected and compensated with a US $20 Amazon e-gift card.

## Results

There were no significant differences in gender, age, and race of the participants (n=702) of the study. Most college students (563/702, 80.2%) participated voluntarily in self-tracking their health using smartphones regardless of disability status (Table 1). More than one-half of the college students with disabilities (51/83, 61%) preferred using a laptop or desktop computer after smartphones, whereas their counterparts leaned toward tablet PCs such as iPads (458/619, 65.2%). In both groups, Health Science majors represented the highest proportion. Among the 83 respondents with disabilities, the following conditions were reported: 28 students (25%) reported having multiple disabilities and a learning disability, followed by autism spectrum disorder (19/83, 23%), hearing loss (18/83, 22%), and physical disability (17/83, 21%). Significant statistical differences were found between the 2 groups when comparing the mean scores of the eHEALS (*t_411_*=–2.22, *P*=.03) and FS (*t_411_*=–4.54, *P*<.001). The group with disabilities showed lower mean scores for both the eHEALS and FS, when compared with the other group.

The main characteristics of self-tracking health practices among the 563 respondents—72 college students with disabilities (86.8%) and 491 college students without disabilities (79.3%)—who reported engaging in self-tracking health are as follows. In the self-tracking health-related questions with allowed duplicate responses, the group with disabilities (34/72, 47%) and the group without disabilities (351/491, 71.5%) responded that they primarily engaged in self-tracking to monitor exercise. Next, the college students with disabilities (29/72, 40%) reported frequently observing their heart rate, while the college students without disabilities (286/491, 53.8%) reported observing their food intake and calorie counting. The group with disabilities engaged in self-tracking health for their physiological health status or medication adherence, while the other group was interested in adopting healthy lifestyle habits (Table 2).

As illustrated in Table 3, the college students were predominantly using smartphones, online software, and wearable devices including fitness trackers and smartwatch as tools for self-tracking health practices. The use of paper and pen was the lowest, accounting for 6% (4/72) and 16.3% (80/491), respectively.

**Table 1:** Comparison of the characteristics between college students with and without disabilities.

Characteristics		College students with disabilities (n=83)	College students without disabilities (n=619)	Total sample (N=702), n (%)	*P* value	
**Self-tracking health, n (%)**						.15
	Yes	72 (86.8)	491 (79.3)	563 (80.2)		
	No	11 (13.2)	128 (20.7)	139 (19.8)		
**Digital device use, n (%)**						<.001
	Laptop/desktop computer	51 (61.4)	454 (73.3)	505 (71.9)		
	Tablet PC	42 (50.6)	416 (67.2)	458 (65.2)		
	Smartphone	55 (66.3)	551 (89)	606 (86.3)		
	Wearable device/fitness tracker/smartwatch	28 (33.7)	231 (37.3)	259 (36.9)		
**Gender, n (%)**						.38
	Male	33 (39.8)	280 (45.2)	313 (44.6)		
	Female	47 (56.6)	313 (50.6)	360 (51.3)		
Age (years), mean (SD)		24.48 (4.38)	24.31 (3.53)	24.33 (3.64)	.69	
Age (years), range		18-33	18-33	18-33	—^a^	
**Race, n (%)**						.053
	White/Caucasian	36 (43.4)	378 (61.1)	414 (59)		
	Black/African American/Hispanic	15 (18.1)	61 (9.9)	76 (10.8)		
	Other	32 (38.6)	180 (29.1)	212 (30.2)		
**Major, n (%)**						<.001
	Computer Science	6 (7.2)	106 (17.1)	112 (16)		
	Business	3 (3.6)	71 (11.5)	74 (10.5)		
	Communications	5 (6)	43 (6.9)	48 (6.8)		
	Government/Political Science	5 (6)	39 (6.3)	44 (6.3)		
	Health Science	18 (21.7)	105 (17)	123 (17.5)		
	Economics	8 (9.6)	93 (15)	101 (14.4)		
	English Language and Literature	6 (7.2)	14 (2.3)	20 (2.8)		
	Psychology	12 (14.5)	46 (7.4)	58 (8.3)		
	Biology	8 (9.6)	21 (3.4)	29 (4.1)		
	Sociology	3 (3.6)	26 (4.2)	29 (4.1)		
	Social Work	2 (2.4)	33 (5.3)	35 (5)		
	Other	6 (7.2)	22 (3.6)	28 (4)		
**Disability, n (%)**						<.001
	Visual impairment	6 (7.2)	—	—		
	Hearing loss	18 (21.7)	—	—		
	Deaf	10 (12)	—	—		
	Learning disability	21 (25.3)	—	—		
	Autism spectrum disorder	19 (22.9)	—	—		
	Physical disability	17 (20.5)	—	—		
	Other	18 (21.7)	—	—		
eHEALS^b^ (possible scores: 8-40), mean (SD)		27.28 (7.32)	28.65 (6.02)	28.65 (6.02)	.03	
eHEALS (possible scores: 8-40), range		11-40	2-10	11-40	—	
FS^c^ (possible scores: 8-56), mean (SD)		35.06 (11.401)	39.99 (8.96)	39.40 (9.41)	<.001	
FS (possible scores: 8-56), range		14-56	15-56	14-56	—	

^a^Not applicable.

^b^eHEALS: eHealth Literacy Scale.

^c^FS: Flourishing Scale.

**Table 2:** Comparative analysis of self-tracking purposes between college students with and without disabilities.

Self-tracking purpose	College students with disabilities (n=83), n (%)	College students without disabilities (n=619), n (%)
Exercise	34 (41.0)	351 (56.7)
Diet/calorie tracking	17 (20.5)	286 (46.2)
Sleep	21 (25.3)	266 (43)
Water intake	23 (27.7)	193 (31.2)
Heart rate	29 (34.9)	193 (31.2)
Blood pressure	19 (22.9)	151 (24.4)
Sedentary time	21 (25.3)	149 (24.1)
Body weight	9 (10.8)	136 (22)
Blood glucose	9 (10.8)	114 (18.4)
Personal health	20 (24.1)	113 (18.2)
Body temperature	17 (20.5)	93 (15)
Stress	11 (13.3)	76 (12.3)
Menstrual cycle	15 (18.1)	74 (12)
Mood	4 (4.8)	71 (11.5)
Medication	17 (20.5)	71 (11.5)

**Table 3:** Comparative analysis of preferred self-tracking tools between college students with and without disabilities.

Self-tracking tools	College students with disabilities (n=83), n (%)	College students without disabilities (n=619), n (%)
Smartphone app	36 (43.4)	324 (52.3)
Online program	30 (36.1)	244 (39.4)
Wearable device	25 (30.1)	210 (33.9)
Computer software	16 (19.3)	182 (29.4)
Digital scale	25 (30.1)	155 (25)
Medical device	25 (30.1)	153 (24.7)
Pen and paper	4 (0.1)	80 (12.9)

The majority of the college students who participated in the survey reported checking their health-related data on a daily or weekly basis. Of the 72 students with disabilities, 21 (25%) engaged in daily health tracking, while 193 of the 218 students without disabilities (31.2%) conducted daily self-tracking of their health.

**Figure 1:**
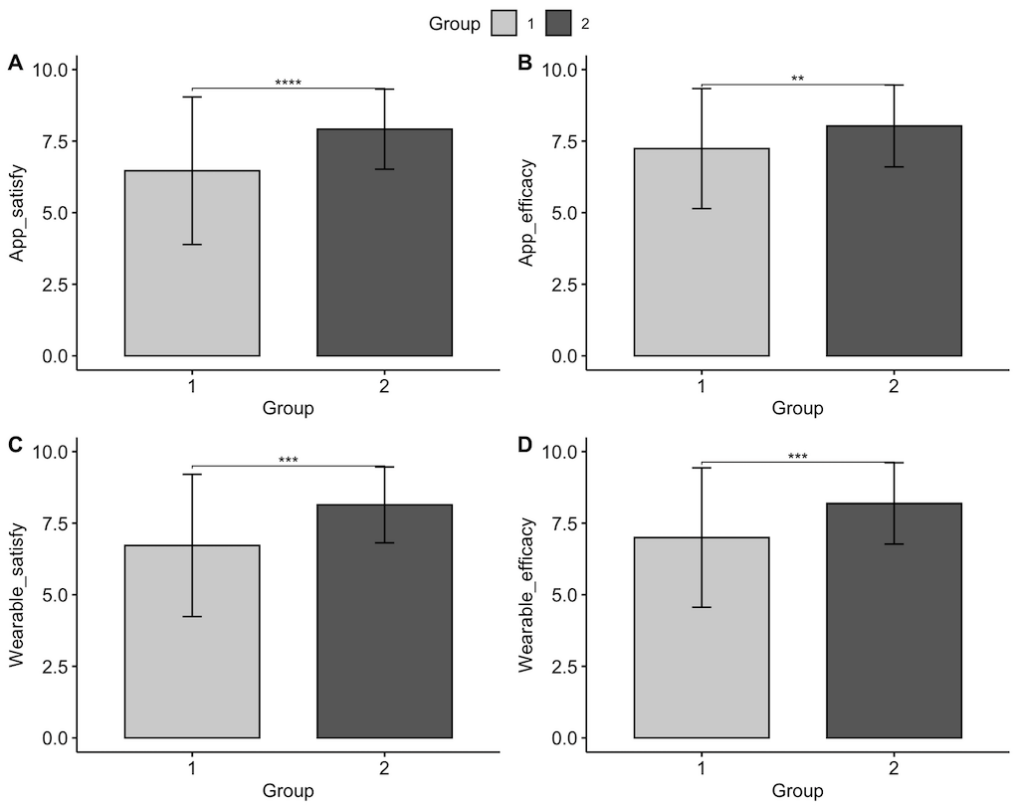
Comparative analysis of (A) satisfaction with health apps, (B) efficacy with health apps, (C) satisfaction with wearable devices, and (D) efficacy with wearable devices between college students with (group 1) and without disabilities (group 2).

To examine whether there were significant differences in satisfaction and perceived efficacy with smartphone health apps and wearable devices based on living with a disability, independent *t* tests were conducted. Satisfaction and perceived efficacy were evaluated on a 10-point Likert scale. The results indicated significant differences based on the presence of a disability in all categories (Figure 1). The satisfaction score for smartphone health apps (*t_411_*=–6.36, *P*<.001) was lower for the group with disabilities (mean 6.47, SD 2.58) compared with the nondisabled group (mean 7.92, SD 1.40), and perceived efficacy (*t_411_*=–3.61, *P*<.001) was also lower for the group with disabilities (mean 7.24, SD 2.10) compared with the group without disabilities (mean 8.03, SD 7.92). The satisfaction score for wearable devices (*t_308_*=–5.97, *P*<.001) was lower for the group with disabilities (mean 6.72, SD 2.48) than the nondisability group (mean 8.14, SD 1.33), and perceived efficacy (*t_308_*=–4.85 *P*<.001) was also lower for the group with disabilities (mean 7.00, SD 2.43) than the group without disabilities (mean 8.19, SD 1.42).

The survey results regarding the reasons why college students who do not engage in self-tracking health provided the following responses. Among college students with disabilities, the top 5 reasons for not engaging in self-tracking health were “I forgot to track” (30/83, 36%), “I don’t think it is useful” (20/83, 24%), “I don’t want to know or see the results” (18/83, 22%), “It is too difficult to track” (17/83, 21%), and “It is not important to me” (13/83, 16%). On the other hand, among college students without disabilities, the primary reasons were “I forgot to track” (221/619, 35.7%), “I don’t have enough time” (190/619, 30.7%), “It is too difficult to track” (130/619, 21.0%), “It is not important to me” (112/619, 18.1%), and “I don’t think it is useful” (90/619, 14.5%).

Finally, to verify the impact of disability status, self-tracking health, and eHealth literacy on college students’ subjective well-being, a multiple linear regression analysis was conducted (Table 4). The multiple linear regression model showed statistically significant results (*F*_1,667_=168.038, *P*<.001), and the explanatory power of the model was 55.7% (*R*^2^=0.557, adjusted *R*^2^ =0.554). Meanwhile, the Durbin-Watson statistic showed a value of 1.93, which is close to 2, indicating that there were no issues with the independence assumption of the residuals. The variance inflation factor was also below 10, indicating that there were no problems with multicollinearity. The significance test of the regression coefficients showed that living with a disability (β=3.81, *P*<.001), self-tracking health (β=2.22, *P*=.03), and eHealth literacy levels (β=24.29, *P*<.001) all had significant positive relationships with overall subjective well-being.

**Table 4:** Relationships between disability status, self-tracking health, eHealth literacy, and subjective well-being in college students, with the Flourishing Scale score as the dependent variable.^a^

Independent variable	B	SE	*t* (*df*)	*P* value	VIF^b^
Constant	3.36	2.52	1.33 (667)	.18	—^c^
Age	–0.05	0.07	–0.80 (667)	.42	1.04
Female gender	–0.29	0.49	–0.60 (667)	.55	1.02
Living with a disability	–2.86	0.75	–3.81 (667)	<.001	1.03
eHEALS^d^	1.08	0.04	24.29 (667)	<.001	1.26
Self-tracking health	1.48	0.67	2.22 (667)	.03	1.24

^a^*F*_1,5_=168.03 (*P*<.001), *R*^2^=0.557, adjusted *R*^2^=0.554, D-W=1.93.

^b^VIF: variance inflation factor.

^c^Not applicable.

^d^eHEALS: eHealth Literacy Scale.

## Discussion

### Principal Findings

This exploratory study utilized an online survey method to examine the self-tracking health practices among college students with and without disabilities. A comparison was conducted with college students without disabilities to identify different patterns. Additionally, the study investigated the correlations between disability status, self-tracking health practices, eHealth literacy, and subjective well-being. The main findings of the study were as follows: (1) irrespective of disability, a majority of college students voluntarily engaged in self-tracking health; (2) college students with disabilities demonstrated significantly lower levels of eHealth literacy and subjective well-being compared with their peers without disabilities; (3) college students with disabilities reported significantly lower satisfaction and perceived efficacy when using smartphone health apps and wearable devices; (4) a significant portion of college students experienced frequent lapses in self-tracking health activities, along with difficulties in using self-tracking methods and interpreting personal health data; and (5) subjective well-being in college students was found to be significantly correlated with disability status, self-tracking health practices, and eHealth literacy.

Many college students reported engaging in digital self-tracking, stating that they check their personal health data on a daily or weekly basis. On the other hand, college students who indicated not engaging in self-tracking health mentioned difficulties in understanding how to track and interpret their health data as reasons for not self-tracking health. The perception of mobile or digital technology being challenging to use was identified as a significant barrier to adopting or maintaining its use. In other words, the complexity of self-tracking digital health technology was identified as one of the main reasons why college students found it challenging to incorporate them into their daily lives, contradicting previous research that suggests the younger population has higher technology familiarity [29]. This finding challenges the assumption that college students, as younger users, are inherently familiar with recent technologies, can easily learn to use them, and may not recognize any hidden usability issues.

Some college students displayed a reluctance to know or address their health status, reflecting a similar trend observed in older adults who avoid self-tracking health due to a heightened awareness of their illnesses and medical symptoms [30]. This means that, when individuals become aware of their existing health problems, collecting health data solely for the purpose of maintaining chronic conditions rather than promoting overall health can create self-avoidance or excessive burden. Sometimes, excessive self-tracking routines can result in adverse effects by making individuals too sensitive to minor fluctuations in health indicators or encouraging them to overinterpret the changed health data. It can also frequently evoke negative feelings for individuals who are diagnosed with chronic health problems. Therefore, it is advisable to avoid unnecessary data visiting for specific health issues. Rather than relying on generalized feedback, it would be more appropriate to offer personalized data-driven feedback that informs self-trackers when a health problem is expected or when immediate health behavior changes are necessary.

It is important to consider incorporating educational features in health-related apps that help college students learn how to monitor and interpret personal health data from a health literate approach [31]. This can include the use of charts, graphics, and illustrations to enhance comprehension [32]. In the cases with visual impairments, a detailed audio description should be added to the visualized health information [33]. Furthermore, as forgetting to engage in self-tracking health practices was identified as one of the primary reasons why college students find it challenging, incorporating time cueing features in the app that align with their daily routines would be beneficial [34]. To assist college students, reminders can be customized to their campus activities or sent at suitable times to notify them when health data collection is incomplete. The most convenient solution for individuals who forget to capture personal health data would be to automatically collect health data through digital devices that they can wear or carry. However, there is an argument that fully automated tracking requires additional feedback to encourage engagement and manage the high volume of data [35]. Therefore, finding a balanced point between manual and automated tracking is necessary.

College students with disabilities reported similar levels of daily use and interest in digital health technology as college students without disabilities. However, their eHealth literacy and subjective well-being levels were significantly lower than for their counterparts. This could be attributed to the direct impact of disabilities, but it could also be interpreted as the result of the benefits they are unable to enjoy due to their disabilities. For example, the college students with disabilities in this study reported significantly lower satisfaction and perceived efficacy with using smartphone health apps and wearable devices. These unsatisfying technology experiences can affect their motivation to engage in self-tracking health practices. This poor user experience for college students with disabilities represents a lack of inclusivity in the current distributed digital health technology, similar to how older adults may feel resistant toward new technology [36]. The fact that users with physical, sensory, and learning limitations cannot experience the same level of positive effectiveness and behavioral changes as common users can be understood as a paradoxical effect of technology that contributes to health inequality in the future [37].

Currently, there are very limited efforts to enhance the usability of mobile or digital health technologies for users with disabilities by addressing their unmet needs, challenges, and barriers. One of the important missing pieces in this study is the exploration of accessibility issues faced by college students with disabilities and their adaptive strategies to overcome these challenges. Recently, Lee and colleagues [38] reported on multiple unique accessibility challenges faced by visually impaired individuals in digital self-tracking systems. They also discussed design opportunities to reduce the gap between visually impaired and sighted users. This highlights the need for greater inclusivity in the design of digital health technologies to ensure that all individuals, regardless of ability, have equal access to the benefits of self-tracking health. Given the heterogeneous nature of disability impacts on technology adoption and users’ preferences and values in technology, diverse disability groups’ user experiences should be deeply understood and considered for future product designs. To prevent the digital divide and its impact on health disparities, a mindset of accessibility and inclusivity is necessary for all software developers and health informatics researchers.

Developing healthy lifestyle habits and the ability to sustain them in the long term is particularly important for college students with disabilities. They are more likely to face barriers and challenges in self-care compared with individuals without disabilities [39,40]. College students with disabilities should receive health education that is tailored to their specific needs and provides them with a proper understanding of health and how to model healthy behaviors. This education should also include information about health information accessibility and the use of necessary assistive technologies to ensure that students with disabilities can freely access and utilize health-related information. For instance, for visually impaired female students, it is important to provide health education on cervical cancer vaccination and breast self-examination in an accessible manner. This could include providing detailed audible descriptions or tangible education materials that they can feel and touch. Ensuring their participation in diverse virtual and in-person health education opportunities should be established.

### Implications

This study identified positive correlations between disability status, self-tracking health practices, eHealth literacy, and subjective well-being among college students with and without disabilities. This highlights the potential benefits of using digital health technology on the well-being and quality of life of all college students. Based on these findings, higher education institutions can consider offering learning opportunities specifically for college students with disabilities, who face additional challenges in managing their health conditions. These opportunities should aim to teach them how to effectively and systematically use digital health technology to monitor their daily health conditions and manage their health behaviors. Experiencing rewarding outcomes from self-tracking their health using digital health technology could lead to improved quality of life and well-being in adulthood.

### Limitations

This cross-sectional, online survey study has certain limitations that warrant careful consideration when interpreting the findings. First, the sampling strategy used in the study was not a sophisticated design. Due to the small sample size of the target population and practical barriers in implementing random or quota sampling, the respondents may not adequately represent the broader population of college students with disabilities. Second, a substantial number of college students majoring in Health Science participated in the survey due to the relevance of the topic to their field of interest, potentially introducing bias to the results. Last, the study did not account for the detailed characteristics of disabilities, such as their type and severity, when interpreting the results. Conducting a subgroup analysis to address this issue would have been ideal; however, the small sample size made it infeasible. Therefore, further research is necessary to obtain study results that reflect the specific characteristics associated with each disability.

### Conclusions

This study indicates that college students with disabilities are engaged in digital self-tracking health activities, much like their peers without disabilities. However, they experience poor satisfaction and efficacy from current digital health technology. Software developers and health informatics researchers should not assume that young adults, including those with disabilities, will readily adopt and effectively utilize technology for health management. Rather, it is crucial to closely examine their abilities and accessibility needs to ensure that digital self-tracking health tools are inclusive and promote long-term self-care. In addition, given their lack of eHealth literacy skills, it is important for health educators in campus settings to provide personalized health consultations for both college students with and without disabilities to enhance the effective utilization of self-tracking health. For college students with disabilities, accessible self-tracking tools can be helpful for establishing healthy behavioral patterns in young adulthood and preventing chronic illnesses, thereby optimizing their life satisfaction and self-control.

## Abbreviations

CHERRIES: Checklist for Reporting Results of Internet E-Surveys
eHEALS: eHealth Literacy Scale
FS: Flourishing Scale
WHO: World Health Organization

## Data Availability

The data sets generated during and/or analyzed during this study are available from the corresponding author on reasonable request.
